# Factors contributing to pre-treatment loss to follow-up in adults with pulmonary tuberculosis: a qualitative evidence synthesis of patient and healthcare worker perspectives

**DOI:** 10.1080/16549716.2022.2148355

**Published:** 2022-12-22

**Authors:** Mercy Namuma Mulaku, Bruce Nyagol, Eddy Johnson Owino, Eleanor Ochodo, Taryn Young, Karen R Steingart

**Affiliations:** a Centre for Global Health Research, Kenya Medical Research Institute, Kisumu, Kenya; b Centre for Evidence-based Health Care, Division of Epidemiology and Biostatistics, Faculty of Medicine and Health Sciences, Stellenbosch University, Cape Town, South Africa; c Department of Pharmacy, Faculty of Health Sciences, University of Nairobi, Nairobi, Kenya; d Honorary Research Fellow, Department of Clinical Sciences, Liverpool School of Tropical Medicine, Liverpool, UK

**Keywords:** Tuberculosis, pre-treatment loss to follow-up, qualitative evidence synthesis, healthcare system, TB care cascade, people-centred care

## Abstract

**Background:**

Since 2018, over 14 million people have been treated for tuberculosis (TB) globally. However, pre-treatment loss to follow-up (PTLFU) has been shown to contribute substantially to patient losses in the TB care cascade with subsequent high community transmission and mortality rates.

**Objective:**

To identify, appraise, and synthesise evidence on the perspectives of patients and healthcare workers on factors contributing to PTLFU in adults with pulmonary TB.

**Methods:**

We registered the title with PROSPERO (CRD42021253212). We searched nine relevant databases up to 24 May 2021 for qualitative studies. Two review authors independently reviewed records for eligibility and extracted data. We assessed methodological quality with the Evidence for Policy and Practice Information Centre tool and synthesised data using the Supporting the Use of Research Evidence framework. We assessed confidence in our findings using Confidence in the Evidence from Reviews of Qualitative Research (GRADE-CERQual).

**Results:**

We reviewed a total of 1239 records and included five studies, all from low- and middle-income countries. Key themes reported by patients and healthcare workers were communication challenges among healthcare workers and between healthcare workers and patients; knowledge, attitudes, and behaviours about TB and its management; accessibility and availability of facilities for TB care; and human resource and financial constraints, weakness in management and leadership in TB programmes. Patients’ change of residence, long waiting times, and poor referral systems were additional factors that contributed to patients disengaging from care. We had moderate confidence in most of our findings.

**Conclusion:**

Findings from our qualitative evidence synthesis highlight multiple factors that contribute to PTLFU. Central to addressing these factors will be the need to strengthen health systems and offer people-centred care.

## Background

Tuberculosis (TB) remains a major cause of morbidity and mortality in many low- and middle-income countries (LMICs) [1]. Despite numerous efforts, resources, and research directed towards TB, these endeavours have not translated to sufficient improvement in health outcomes. According to the World Health Organization (WHO), TB incidence has been decreasing globally at a rate of 2% per year; however, the reduction was not fast enough to reach the 2020 milestone of the WHO End TB Strategy [2,3]. The global targets included a reduction in TB incidence and mortality of 20% and 35%, respectively, from 2015 to 2020. However, in 2020, these indicators fell short at 11% and 9% respectively, with the incidence being around 50% and mortality around 25% of the way to the 2020 milestone. Africa has been making good progress with reductions of 19% (incidence) and 18% (mortality), though these percentages are still below the 2020 milestone [3]. Sustaining these improvements will be challenging because owing to the COVID-19 pandemic, globally, deaths from TB are increasing for the first time in a decade [3].

Delays in diagnosis and initiation of effective treatment contribute to challenges in TB care [3,4]. Therefore, access to rapid diagnostics and treatment will play a key role in reducing morbidity and mortality. The TB care cascade described by Subbaraman, and colleagues demonstrated that many patients are lost to follow-up after being diagnosed with TB, but before starting treatment [5]. These patients continue to spread TB in the community and most die because of the disease [6]. In a systematic review that included 23 studies, MacPherson and colleagues found that the overall percentage of pre-treatment loss to follow-up (PTLFU) ranged from 4% to 38%, with studies from Africa ranging from 6% to 38% [6].

Given that many people diagnosed with TB experience PTLFU, it is important to understand the factors that contribute to this attrition to come up with effective interventions. Several studies have highlighted reasons that lead patients to drop out before starting treatment without looking at the challenges experienced during the linkage to care [5–7]. Quantitative research suggests that patients face healthcare system barriers that may interfere with their receiving care leading to PTLFU [6–8].

There are limited qualitative data to provide more insight into PTLFU. Therefore, conducting this qualitative evidence synthesis (QES), where we systematically searched for qualitative primary studies and summarised their findings, will assist in generating comprehensive evidence on contributors to PTLFU that goes beyond the findings of a single study. The QES will give an understanding of how different factors and contexts influence PTLFU through the perceptions of patients and healthcare workers (HCWs). This in turn will guide us in coming up with effective approaches to reduce PTLFU. To our knowledge, no other QES on perspectives and experiences of patients and HCWs on contributing factors to PTLFU has been conducted so far.

The objective of this QES was to identify, appraise, and synthesise evidence on the perspectives and experiences of patients and HCWs on factors contributing to PTLFU in adults with pulmonary TB.

## Methods

We registered the title with the International Prospective Register of Systematic Reviews (PROSPERO): CRD42021253212 and we published the protocol in the open science framework [9]. We have reported this QES according to the Enhancing transparency in reporting the synthesis of qualitative research ENTREQ statement [10].

### Criteria for considering studies for the QES

We included primary studies that used qualitative study methodology to describe the experiences and perspectives of patients (adults aged ≥18 years with pulmonary TB) and HCWs on PTLFU and studies that focused on PTLFU. We defined PTLFU as people in a national TB care programme who received a diagnosis of TB based on at least one positive smear, culture, or molecular WHO-recommended rapid diagnostic test (mWRD) but did not start TB treatment including those who died before starting treatment [6,8,11]. We included studies that used both qualitative methods for data collection (e.g. focus group discussions, individual interviews, observation, brainstorming, open-ended survey questions) and qualitative methods for data analysis (e.g. thematic analysis, framework analysis, grounded theory). For studies that used mixed methods, we included only data that had been collected and analysed using qualitative methods. We included studies from all geographical settings. We excluded studies that collected data using qualitative methods but did not analyse the data using qualitative analysis methods (e.g. open-ended survey questions where the response data were analysed using descriptive statistics only).

### Identification of studies

We conducted a comprehensive literature search in the following databases: MEDLINE (Ovid from 1946), Cochrane Library (Issue 5 of 12 May 2021), EMBASE (Ovid from 1947), CINAHL (Cumulative Index to Nursing and Allied Health Literature), Global Index Medicus, LILACS, HERDIN, Science Citation Index and Social Science Citation Index. We performed the search up to 24 May 2021 without date restriction. We only searched for studies written in English since we did not have resources to support the translation of non-English language studies. A detailed description of the identification of studies and full search strategy can be found in Supplemental material 1. We entered the search output into EndNote to delete duplicates. Thereafter, we used Covidence, an online platform for systematic reviews for screening and study selection [12].

### Selection of studies

MM with either BN or EJO independently screened titles and abstracts for relevance. Thereafter, we obtained full texts for the selected titles and abstracts. MM with either BN or EJO independently assessed full texts against eligibility criteria using Covidence to come up with the final list of the included studies [12]. We documented the reasons for excluding the studies. We resolved any disagreements that arose at each stage through discussion and where necessary by consultation with a third review author (KRS).

### Data collection and management

We extracted data using a structured format, the Supporting the Use of Research Evidence (SURE) framework, to retrieve information [13]. To ensure validity, three review authors (MM, BN, EJO) piloted a predesigned form by extracting data from three (60%) of the included studies. We modified the form based on the pilot. Thereafter, one review author (MM or BN, or EJO) extracted data from the included studies using the finalized form and a second review author (MM) verified the information. We resolved disagreements through discussion and consensus-building. We extracted the following data: first author, date of publication, setting (country, type of health facility, country income level, and TB and HIV burden), study design, type of participant (patient or HCW), method of data collection, type of data analysis (such as thematic and framework), and reported experiences and perspectives. We assigned country income levels according to the World Bank List of Economies [14]. In addition, we classified countries as being a high burden or not a high burden for TB, HIV-associated TB, and multidrug-resistant (MDR)/rifampicin-resistant TB based on the WHO classification for the period 2021–2025 [15].

### Assessment of methodological quality

We assessed the methodological quality of the studies using the Evidence for Policy and Practice Information (EPPI) Centre tool, which has been used in other qualitative reviews [16,17]. We assessed the following domains using the tool: rigour in the sampling, rigour in the data collected, the rigour of data analysis, support of the findings from the data, breadth and depth of the findings, reliability of the study findings, and relevance of the study findings to the aims of the synthesis. Based on predefined criteria provided by the EPPI Centre tool, domains were scored as yes, a fairly thorough attempt was made; yes, several steps were taken; yes, a few steps were taken; and no, not at all/can’t tell. MM, with either EJO or BN, applied the EPPI Centre tool independently and we resolved any disagreements through discussion and consensus-building.

### Data synthesis and analysis

We analysed the data separately for patients and HCWs as we expected to find differences between these two groups. We conducted the synthesis of data using five stages of the thematic framework synthesis as follows [18,19]. One review author (MM) familiarized herself with the data against the QES objective and noted recurrent themes across studies. We used a predetermined thematic framework developed by SURE guidelines to guide the thematic analysis [13]. We adopted the framework based on the emerging themes from the included studies. The framework provided a list of factors that contributed to PTLFU and possible strategies to reduce PTLFU. MM, with either BN or EJO, independently reviewed the data to identify themes. As new themes emerged, we modified the framework. We continued this process through discussion and consensus until there were no new emerging themes. We coded and sorted data based on the themes identified in the primary studies and displayed the themes in an analysis table (Chart). Specifically, we presented studies and related themes in columns and rows of the table, thereby enabling the review authors to compare the findings of the studies across different themes and subthemes. We explored associations between themes to help clarify our findings. We mapped and interpreted the findings by the QES objective and emerging themes.

### Assessing our confidence in the QES findings

We used GRADE-CERQual (Confidence in the Evidence from Reviews of Qualitative Research) to assess confidence in the QES findings [20]. GRADE-CERQual includes four key components: methodological limitations of included studies; coherence of the QES findings; adequacy of the data contributing to the QES finding; and relevance of the included studies to the QES question. MM initially assessed the confidence in each QES finding and EO checked her assessment, resolving any disagreements by discussion. We classified overall confidence as high, moderate, low, or very low. We presented the CERQual assessment and explanations in a Summary of findings table.

## Results

### Study selection

**Figure 1.**
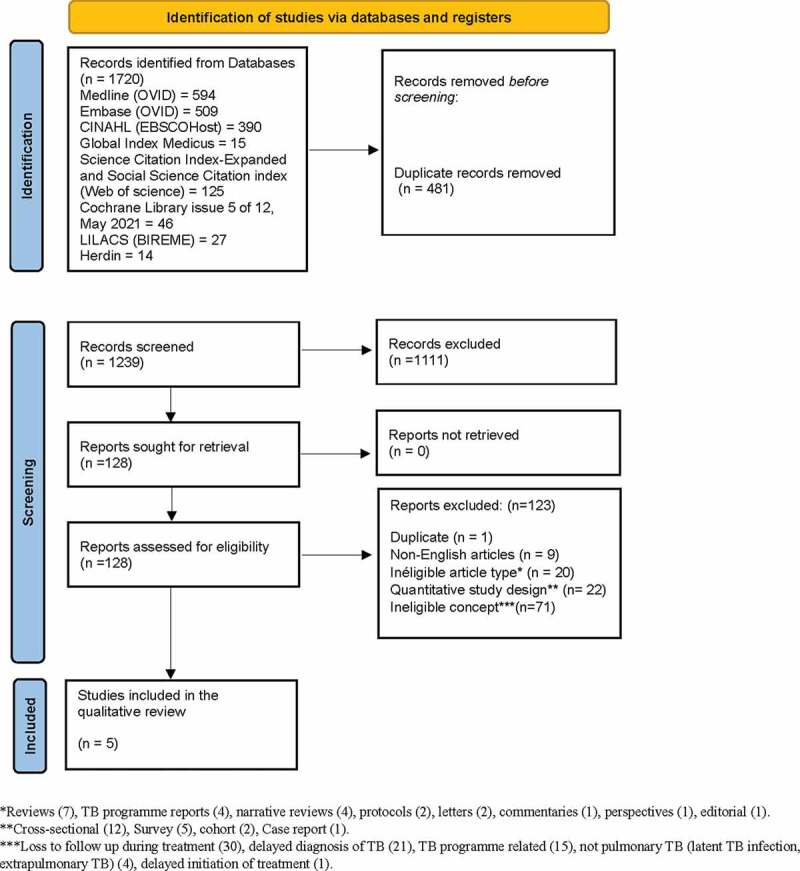
PRISMA flow diagram.

We identified a total of 1720 records through database searching. After removing the duplicates, we screened 1239 records by title and abstract to remove irrelevant reports and excluded 1111 records. We retrieved 128 reports and, after assessing them against the inclusion and exclusion criteria, excluded 123 reports (see Supplemental material 2). The main reason for exclusion was the ineligible concept (n = 71), which we described further as the loss to follow-up during treatment (n = 30); delayed diagnosis of TB (n = 21); TB programme related (n = 15); not pulmonary TB (i.e. latent TB infection, extrapulmonary TB) (n = 4); and delayed initiation of treatment (n = 1). We finally included a total of five studies in the QES [21–25] (Figure 1).

### Characteristics of included studies

Of the five included studies, three studies used qualitative methods (critical incident narratives, exploratory and grounded theory) only [21,24,25], while two used a mixed methods approach [22,23]. Three studies were from India [22,23,25], one from Malawi [21], and one from South Africa [24]. The participants were HCWs, TB programme managers, patients who experienced PTLFU, and family members of the patients who experienced PTLFU and had died. The studies were in both rural and urban settings and included primary, secondary, and tertiary care facilities. Three studies took place in lower-middle-income settings [22,23,25], one study in an upper-middle-income setting [24], and one study in a low-income setting [21]. All studies were from high TB/HIV burden countries while four of the studies were from high TB and MDR-TB burden countries [22–25] Table 1.

**Table 1.** Characteristics of included studies in the qualitative evidence synthesis for pre-treatment loss to follow-up.

Author, year of publication	Country, setting	Study aims	Study design	Participants (number); sex (number, %)	Data collection methods	Data analysis methods
Squire 2005	Malawi Low-income High TB/HIV burden Urban District hospitals, health centres, public laboratories	To locate smear-positive pulmonary tuberculosis patients who were identified during the first 6 months of 2000 but did not start treatment (‘lost cases’) To describe these patients’ pathways to diagnosis, health status, and socio-demographic characteristics To explore why these patients did not start treatment	Qualitative, critical incidents narrative interviews	Patients (n = 19); female (n = 10, 53%), male (n = 9, 47%) HCWs (n = 46)	Focus group discussion Key informant interviews	Frame-work analysis
Sharma 2017	India Lower middle income High TB, TB/HIV, and MDR-TB burden Rural, peri-urban, and urban Chest clinics equivalent to District TB centres	To examine the various reasons for pre-treatment loss to follow-up among new sputum-positive cases diagnosed under the Revised National TB Control Programme in Delhi To propose an intervention model to reduce pre-treatment loss to follow-up based on the provider’s feedback and the health-seeking behaviour of patients	Mixed methods, qualitative methods used exploratory design	Focus group discussion (n = 9): paramedics, laboratory technicians, TB health visitors TB district managers (n = 24); sex not specified	Focus group discussion Brainstorming session	Thematic analysis
Mwansa-Kambafwile 2020*	South Africa Upper middle income High TB, TB/HIV, and MDR-TB burden Johannesburg (urban)	Exploring reasons for TB’s initial LTFU from the perspectives of TB programme managers and outreach WBOT programme managers, with a focus on the WBOT’s (potential) role in reducing initial LTFU	Qualitative, exploratory design	Healthcare managers (n = 9); female (n = 6, 67%), male (n = 3, 33%)	In-depth interviews	Frame- work analysis
Stalin 2020	India Lower middle income High TB, TB/HIV, and MDR-TB burden Peripheral health Institutions	Measure the effectiveness of the new intervention package, developed based on a qualitative study in reducing PTLFU of all (TB) patients diagnosed and referred for treatment from medical colleges to peripheral health institutions	Mixed methods, qualitative, exploratory design	Patients, HCWs, laboratory workers, healthcare managers, TB health visitors (n = 34); sex not specified	In-depth interviews	Thematic analysis
Thomas 2020	India Lower middle income High TB, TB/HIV, and MDR-TB burden Rural, urban National Tertiary Referral and Teaching Hospitals Primary and secondary health facilities	To analyse qualitative data on PTLFU from TB patients and HCWs in Chennai, one of India’s largest cities	Qualitative-grounded theory	Patients (n = 20), health visitors (n = 18), laboratory workers (n = 17), senior treatment supervisors (n = 18), and family members of PTLFU patients who had died (n = 13) Patients (n = 33); females (n = 3, 9%), males (n = 30, 91%) HCWs (n = 40); females (n = 10, 25%), males (n = 30, 75%)	Focus group discussion, In-depth interviews	Thematic analysis

**Abbreviations: HCWs**: healthcare workers; **LTFU**: loss to follow-up; **PTLFU**: pre-treatment loss to follow-up; **TB**: tuberculosis; **WBOT**: Ward-based Outreach Team.

*The publication refers to initial loss to follow up though the definition is similar to PTLFU, that is why we included the paper.

### Assessment of methodological quality

All studies had taken several steps in ensuring there was rigour in sampling in that there was variation in the participants who were interviewed. Three studies did not report detailed demographic characteristics for their participants [21–23]. Regarding rigour in data collection, four studies had taken several steps in ensuring data collection tools were well prepared, informed consent was conducted with the participants, and more than one method of data collection was used. Only one study reported piloting the data collection tool [24]. One study indicated there was written informed consent, but the process was not described [22]. One study reported on the duration of the interviews [25]. Rigour in data analysis was fairly well done in most of the studies with two of the studies making a thorough attempt [24,25]. These two studies described their data analysis methods in detail and had a second person review the data to ensure that the information provided by the participants was accurately captured. One study had insufficient participant quotes, and these were not coded to support the themes that arose during the brainstorming session and the focus group discussion [24]. Study findings were adequately supported by the data (participants’ quotes) in three studies [21,24,25]. In terms of depth and breadth of study findings, in one study, the authors made a thorough attempt at exploring different reasons for PTLFU, going beyond the descriptive data provided by the participants to come up with an explanatory model [25]. One study had limitations when it came to breadth and depth in that the authors included few quotes to support the findings, and these were mainly from the patients who experienced PTLFU [23]. The study had only two quotes from the HCWs to support their perspectives, (Supplemental material 3). We have provided a detailed assessment in Supplemental material 4.

### QES findings

**Figure 2.**
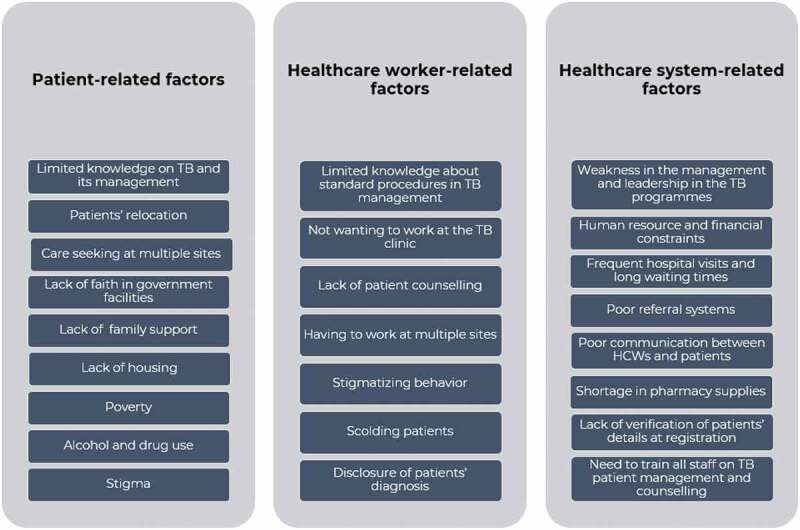
Summary of factors contributing to pre-treatment loss to follow-up.

We grouped the factors contributing to PTLFU into those related to patients, HCWs, and the healthcare system (Figure 2).

### QES themes

Themes related to patients are described in Table 2, HCWs in Table 3, and the healthcare system in Table 4.

**Table 2.** Representative illustrations of patient-related factors contributing to pre-treatment loss to follow-up.

Main theme	Subtheme	Illustrations from the studies
Patients’ knowledge, attitudes and behaviours about TB and its management	Knowledge about TB and its management	*Q1. “Some patients feel that TB is a result of ‘‘bad’’ air. So, to have a ‘change in environment, they visit Vrindavan (Old TB sanatorium faith healers exist in Vrindavan) for fresh clean air.”* (Brainstorming session of programme managers, India).
	Patients’ relocation	*Q2. Patient relocation emerged strongly as a reason. ‘ … . patients are always relocating.’* (Programme manager, South Africa).
	Wrong contact address	*Q3. ‘Patients give wrong phone numbers and wrong addresses. Unfortunately, this is only discovered at the point of defaulter tracing.’* (TB programme managers, South Africa).
	Care seeking at multiple sites ‘Shopping around’	*Q4. ‘They like to confirm, and they will come here and test and if they are positive here, they will go to another facility to test. Maybe if still not satisfied, they will go to another.’* (Programme manager, South Africa).
	Lack of faith in government healthcare facilities	*Q5. ‘Patients did not have faith in the government-run health system and doubted the quality of free drugs available at the DOT centres.’* (Brainstorming session of TB programme managers, India).
	Alcohol and drug use	*Q6.* “*They think, ‘if we take tablets, we can’t drink alcohol, so just to continue alcohol, they don’t want to take drugs …* .” (TBHV, India).
Patients’ motivation to attend TB appointments	Lack of family support	*Q7. ‘Sometimes patients get worse after testing, get admitted to hospital and therefore fail to return for the results. It is important that a relative or friend informs the facility so that the patient is not labelled as LTFU*.’ (Programme manager, South Africa).
	Stigma	*Q8. ‘These days whenever the patient has been diagnosed as TB patient, they always suspect that he or she is HIV positive.’* (FGD, 2, Malawi).
		*Q9. “I came here without my family members’ knowledge, as they otherwise would not allow me to visit (this TB specialty hospital). If others learn that I am visiting (this hospital), they will tag me as being a TB patient.”* (Woman without a prior treatment history, India).
	Lack of housing	*Q10. “We don’t have a house; we lived on the platform (Indian term for homelessness) for 9 months*. (Family member of a man without a prior treatment history, India).
	Poverty	*Q11. ‘Costs mentioned included transport, food, daily necessities, and, in the case of admission in hospitals, medical expenses such as drugs*.’ (Female patient, Malawi).

**Abbreviations: FGD**: Focus group discussion; **HCW**: healthcare workers; **LTFU**: loss to follow up; **TB**: tuberculosis; **TBHV**: TB health visitor.

**Table 3.** Representative illustrations of healthcare worker-related factors contributing to pre-treatment loss to follow-up.

Theme	Subtheme	Illustrations from the studies
HCWs’ knowledge, attitudes, and behaviours about TB and its management (*reported by HCWs)*	Knowledge about TB and its management	*Q12. ‘Patient is sputum positive but doesn’t receive treatment.’* (FGD of TBHVs and LTs India). *Q13. ‘They give the sputum [the sputum sent to the laboratory] the outcome, they say it is negative, but the patients still cough … and we ask him to give us again we do the same, they found him negative, and we tell him do not be annoyed give us sputum sample for the third time and we took to the lab they say it is negative.’* (FGD of HCWs, Malawi).
	HCW attitudes towards their work	*Q14. ‘Participants reported that staff generally do not like to work in the TB room and are reluctant to acquaint themselves with systems and processes on TB patient management’* (TB programme managers, South Africa).
		*Q15.* Senior Treatment Supervisor 4: ‘*Few patients don’t return, so we can’t do anything more (to retrieve them).’* Moderator: ‘*So, you never register that case?’* Senior Treatment Supervisor 5: ‘*Yes, we never register that case.’* (Excerpt from a Senior Treatment Supervisor FGD, India).
	Lack of patient counselling	*Q16.* ‘*I think it’s probably … patients are not counselled when they are tested … you know like in HIV. So, the patients do not know the importance of starting treatment soon. So, they just go home. And remember they test and are given a date when to come back and this is where the gap is. They normally don’t come back on their own*.’. (Programme manager, South Africa).
	Stigmatizing behaviour	*Q17. ‘Doctors don’t have enough time to talk to patients. The staff nurse can counsel patients, but they treat patients as untouchables.’* (Participant in a health visitor FGD, India).
	Disclosure of the patient’s diagnosis	*Q18. ‘(When the TB diagnosis has been disclosed), patients will ask us, “why did you share my information with the (community)leader?… Who gave you the right to share information regarding my disease condition with others? What will others think about me?”.’ (*Participant in a health visitor FGD describing the type of; language used by patients after disclosure of diagnosis, India).
HCWs’ attitudes and behaviours about TB and its management (*reported by patients*)	Stigmatizing behaviour	*Q19. ‘They (staff in the hospital) treated me in an irreverent way.’ (*Family member of a man without a prior treatment history, India).
	Scolding	*Q20. ‘Some of (the HCWs) talked nicely to me, but the doctor scolded me, so I didn’t return to pick up my test result*.’ (Woman without a prior treatment history, India).

**Abbreviations: DMCs**: Designated microscopy centres; **FGD**: Focus group discussion; **HCWs**: healthcare workers; **TB**: tuberculosis.

**Table 4.** Representative quotations on healthcare system-related factors contributing to pre-treatment loss to follow-up.

Theme	Subtheme	Illustrations from the studies
Accessibility and availability of facilities for TB care	Frequent hospital visits	*Q21. ‘Many patients are not able to come to the hospital several times, especially old people. It will be very helpful if the number of required visits is reduced.’* (Woman without a prior treatment history, India).
	Long distance	*Q22. “Patients were not referred to the correct PHIs where they reside. Therefore, the distance of DOTS centres from patients’ houses was long which made them inaccessible.”* (India).
	Long waiting times	*Q23. ‘* … *give excuses that they are long queues.’* (WBOT district coordinator, South Africa). *Q24. ‘ … many patients reported of unpleasant experience at medical college hospital such as … . long waiting period….’* (Patients, India).
	Lack of pharmacy supplies	*Q25. ‘There was also unavailability of drugs at the time of reporting to the referred DOTS centre. Patients were asked to come back after some time, but they did not report back*.’ (Patient, India).
Human resource and financial constraints	Costs HCWs incurred	*Q26. ‘For phone calls (with patients or other HCWs) we are spending almost 1000 rupees per month from our own pocket*.’ (Participant in a Senior Treatment Supervisor FGD, India).
	Lack of reimbursement for the health workers (costs incurred out of pocket)	*Q27. ‘Most villages do not have any buses … those of us who have two-wheelers make these visits (to track PTLFU cases), but we are not receiving any reimbursement for petrol costs.’* (Participants in a health visitor FGD, India).
	High workload	*Q28. ‘We need manpower … one Laboratory Technician is working at four DMCs. If I collect the first sputum of the patient today (Friday), I will get a chance to collect the second sputum of that patient only on the next Friday.’* (Participant in a Laboratory Technician FGD, India).
	Frequent staff rotation	*Q29. ‘Participants suggested less staff rotation in the TB room so that continuity of optimal TB patient care is guaranteed*.’ (TB programme manager, South Africa).
	Teamwork among the PHC in the RNTCP	*Q30. ‘ … And then if someone comes to stand in for the TB sister, they will not attend to the patient thoroughly as they’re supposed to, sometimes not even informing them about the importance of ensuring they come back for the results*.’ (Programme manager, South Africa).
Challenges in internal and external communication in the TB healthcare facilities	Poor referral systems	*Q31. ‘They didn’t inform me properly about the disease. I was referred to a hospital where they told that I was supposed to go to some other hospital which is quite far from home….’ (Male patient, India).*
	Lack of and poor communication between HCWs and the patients	*Q32. ‘They referred me to a hospital for treatment … But they did not say anything about my health condition and my disease*.’ (Man without a prior treatment history, India).
		*Q33. ‘Many patients said that they did not report due to their unpleasant experience at medical college hospital such as harsh comments by health staff, long waiting period and no proper briefing and counselling about the disease.’* (Patients, India).
		*Q34. ‘Non-cooperative staff at DOT centres, poor patient-provider interaction, poor communication skills of TBHV/LT*.’ (Brainstorming session of TB programme managers, India).
Education and training	Training on TB, patient management, and counselling services	*Q35. ‘Participants suggested less staff rotation in the TB room and TB patient management education to all facility clinical staff so that continuity of optimal TB patient care is guaranteed*.’ (TB programme managers, South Africa).
Management and leadership in the TB programme	Limitations in the HCW’s mandate	*Q36. ‘The WBOTs cannot refer the patients to their team leader, if the latter is an enrolled nurse, for treatment initiation because enrolled nurses cannot prescribe medications in the South African healthcare system*.’ (Programme manager, South Africa).
	Teamwork	*Q37. ‘There are some issues when TBHV is on leave. Other staffs are busy with their own works. They are not willing to take up additional responsibilities.’* (Senior treatment supervisor, India).
	Lack of respect for junior staff	*Q38. ‘I can’t go and talk to the ward patient when he is available, because, at the same time, I will be called by the medical officer to do other work … I can’t tell the doctors about the challenges I face.’* (Participant in a Health Visitor FGD, India).
		*Q39. ‘They call on me while I’m trying to do my work … . They send someone to get me saying, “call that RNTCP girl”.’* (Participant in a health visitor FGD, India).

**Abbreviations: DOTS**: Directly Observed Treatment centres; **FGD**: Focus group discussion**; HCWs**: healthcare workers; **IDI**: In-depth Interview; **LTFU**: loss to follow up; **PHC**: Primary health centre; **PHIs**: peripheral health institutions; **PTLFU**: pre-treatment loss to follow-up; **RNTCP**: Revised National TB Control Programme; **TB**: tuberculosis; **TBHV**: TB health visitor; **TBHV/LT**: TB health visitor/Laboratory technician; **WBOT**: Ward-based Outreach Team.

### Theme 1: patients’ knowledge, attitudes, and behaviours about TB and its management

Overall, patients reported having limited knowledge about the cause of TB. Although some patients described TB as a gradual deterioration in health accompanied by a persistent cough, others thought the disease was caused by ‘bad air’ (Q1). Some patients were unaware that their symptoms could be related to TB while others associated a TB symptom such as cough with having HIV/AIDS.

‘*They [the community] just take it that this is just coughing until one’s health is deteriorating. They then start developing a suspicion that this may be HIV – this is now becoming the common thing because we have lost so many … most of the people these days assume that once someone has got the coughing that this may be HIV-related coughing* . … ’ (Father of the deceased, Malawi) [21].

Patients’ attitudes and behaviours may have contributed to their experiencing PTLFU. For example, patients relocated and sought care elsewhere without informing the facility where they initially received their diagnosis (Q2). Others gave wrong contact details at the point of patient registration which made tracing and follow-up challenging for HCWs (Q3). In addition, patients may have sought care at multiple sites and been lost to follow-up before collecting test results or receiving a referral for treatment (Q4). And some patients reported that they doubted the credibility of the healthcare institution given that it was a government facility (Q5).

*‘I didn’t get proper treatment at (the first tertiary hospital) due to lack of staff, and I left the (second tertiary hospital) due to lack of hygiene and cleanliness. So, I decided to go to (a third facility) for further care.’* (Man without a prior treatment history, India) [25].

Moreso, people with TB who use alcohol and drugs reported they were concerned that, after starting TB treatment, they would be unable to continue to use these substances (Q6).

#### Theme 2: patients’ motivation to attend TB appointments

Patients mentioned several reasons affecting their motivation to attend TB appointments leading to PTLFU: lack of support from family members (Q7), TB-related stigma (Q8, Q9), lack of housing (Q10), and inability to pay for medical expenses (Q11).

*‘ … MK. 100.00 was not enough so we just decided to buy some medication so that it could help, and I think it took almost one week until she died.’* (Father of the deceased, Malawi) [21].

Additionally, people reported being fearful about receiving TB care in the hospital owing to negative outcomes for other patients.

*‘Yes, the doctor admitted him in the ward … (T)wo patients died close to my husband … the next night two more patients also died there so my husband became very scared and we discharged him.’* (Family member of a man without a prior treatment history, India) [25].

### Theme 3: HCWs’ knowledge, attitudes, and behaviours about TB and its management

HCWs reported having variable knowledge and skills regarding TB and its management. Most HCWs had good knowledge of TB and could define ‘pre-treatment loss to follow-up’ (Q12). Others understood how the TB programme works, especially the protocol for initiating treatment. Conversely, a few HCWs were unclear about TB programme operations and the protocol for initiating treatment.

‘*I … I really don’t know how they work in the TB programme*.’ (WBOT programme manager, South Africa) [24].

Additionally, some HCWs lacked knowledge and skills in managing patients presumed to have TB (Q13). TB programme managers and HCWs described several factors related to HCWs that contributed to PTLFU: having a negative attitude towards their work, such as being reluctant to work in the TB clinics (Q14, Q15) and not counselling patients properly about TB (Q16). Some patients reported feeling stigmatized when HCWs did not spend enough time talking to them (Q17). Consequently, patients did not want to return to the health facility. Some patients refused to seek further care when their diagnosis was disclosed to other healthcare team members (Q18).

*‘We once visited one of the (PTLFU) patients (at home) with our team, including the doctor, Senior Treatment Supervisor, Senior TB Laboratory Supervisor, and Health Visitor. But he said, “I feel ashamed because of your action, so I cannot take medicines”.’* (Participant in a Health Visitor FGD, India) [25].

Patients reported having unpleasant experiences when they visited the health facilities such as the treatment they received from the HCW (Q19) and being scolded by the HCW (Q20).

### Theme 4: accessibility and availability of facilities for TB care

Patients reported that cost and time travelling due to the frequent visits (Q21) and long distance (Q22) to the TB centres dissuaded them from accessing care.

Additionally, patients were discouraged when they reached the health facility and did not receive care due to long waiting times (Q23, Q24), lack of pharmacy supplies (Q25), and failure of the power supply. After encountering these barriers, some patients never returned.

*“Thrice I came to (a tertiary hospital) to receive my test report but … they said, ‘ … you have to wait for some days … We can prepare your report only when the power supply is available.’* (Woman without a prior treatment history, India) [25].

### Theme 5: human resource and financial constraints

HCWs reported having out-of-pocket expenses for which they were not reimbursed (Q26, Q27). These financial constraints limited them from performing their roles effectively. Examples are sending a referral form via courier to the TB care facility, making phone calls, and covering the travel costs required to follow up with patients.

*‘Due to practical difficulties, we never send this column (copy of the referral form). If I have to send this column (back to the Diagnostic Microscopy Centre (DMC)) then I need to spend money from my pocket for purchasing the envelope and paying the courier charge*.’ (Participant in a senior treatment supervisor FGD, India),[25].

Although some HCWs received a travel allowance, they were less inclined to provide follow-up visits with patients because they felt the allowance was inadequate.

*‘Inadequate motivation of staff for conducting default retrieval, long-distance between DOT centre and catchment area, lack of transport facility for health visitor to make retrieval visits. Conveyance allowance for the TB health visitor is considered inadequate by them resulting in decreased motivation of staff for conducting home visits.’* (Brainstorming session of TB programme managers, India) [22].

Regarding human resources, HCWs pointed to the high workload as a factor contributing to PTLFU. Most mentioned that owing to staff shortages they had to work at more than one facility which caused delays between sputum collection and receipt of test results (Q28). Additionally, HCWs were not able to execute their duties as required such as verification of patient contact information, especially those working in high-volume facilities.

*‘It is very difficult (to verify patient contact information) in big centres (i.e. high-volume facilities) because they are regularly overcrowded with patients.’* (Participant in a senior treatment supervisor FGD, India) [25].

Other reasons related to human resources that contributed to PTLFU comprised frequent staff rotation at the TB clinics that disrupted continuity of care (Q29) and lack of teamwork amongst the staff at the TB centres (Q30). This happened mostly when the staff at the TB clinic was on leave.

‘*There are some issues when TB health visitor (TBHV) is on leave. Other staffs are busy with their own works. They are not willing to take additional responsibilities of TBHV* … .’ (Senior treatment supervisor, India) [23].

### Theme 6: challenges in internal and external communication in the TB healthcare facilities

Communication among HCWs and coordination in and between facilities were challenges that contributed to PTLFU. Patients experienced frustration during referrals from one facility to another (Q31).

*‘Doctors in medical college were not in favour of intermittent therapy, therefore after investigations at DMC, they started the patient on treatment but did not provide any feedback regarding treatment to the specific DOT/DMC where the sputum was tested*.’ (FGD of medics and paramedics, India) [22].

Similarly, communication between HCWs and patients was a contributor to PTLFU. After learning that their sputum tested positive for TB, patients were not clear about the next steps and what to do when they were referred from one facility to another (Q32). In addition, HCWs had poor communication and counselling skills (Q33, Q34). Consequently, patients were reluctant to return to the facility when the referral from one clinic to another was frustrating.

*‘We went to (a tertiary hospital) for an initial check-up … . They didn’t tell us much. They said go to number 3 (outpatient clinic) and then number 5 (outpatient clinic) and back again for 2 days. After running from pillar to post, we just gave up and returned home.’* (Family member of a man without a prior treatment history, India) [25].

### Theme 7: education and training

Some HCWs had not been trained in TB management, which contributed to the interruption of TB services when trained TB staff were unavailable (Q35).

In some settings, most of the patients who experienced PTLFU had special needs such as migrants and people who use drugs and alcohol [22,23,25]. However, HCWs lacked the knowledge and skills to reach out to people with special needs.

*‘People who abuse alcohol and drugs have psychological problems and need special counselling …*.’ (FGD of TBHVs and Laboratory Technicians (LTs), India), [22].

### Theme 8: management and leadership in the TB programme

Concerns were voiced regarding the management and leadership of the TB programme which led to PTLFU. Teamwork and capacity to make decisions on TB care were lacking among the HCWs, which consequently affected the delivery of TB health services (Q36, Q37). Additionally, junior staff felt overworked and disrespected when senior staff gave them assignments unrelated to TB care (Q38, Q39). Also, the working hours at the directly observed treatment (DOT) centres (also referred to as treatment support centres) were not people-centred, especially for those who were running businesses and women who had other responsibilities at home.

*‘At the grass-root level, the programme has its functional units as DMCs and DOT centres catering to specific areas. DOT centres located at public health facilities have fixed timings (8 am–2 pm) that may be inconvenient for different sections due to reasons related to working hours.’* (Brainstorming session of TB programme managers, India) [22].

Also, the paperwork during patient referral was not clear, which led to patients dropping out of care when they were sent to the referring facility for more documents.

*After the patient reached the rural DOT centre, the HCW said): ‘Go back to (tertiary hospital where the patient was diagnosed in the city) and bring a referral slip – only then can we start treatment.’* (Man without a prior treatment history, India) [25].

### Confidence in the QES findings

We used GRADE-CERQual to assess our nine review findings. We graded three themes as high confidence and six themes as moderate confidence. The summary of findings is presented in Supplemental material 5. We have provided a detailed assessment including explanations for our grading (see Supplemental material 6).

## Discussion

Our QES found that many factors contribute to PTLFU. Central to addressing these factors will be the need to strengthen health systems and offer people-centred care, which are vital to ensuring good patient outcomes. Reported healthcare system-related factors consist of accessibility and availability of facilities for TB care, human resource and financial constraints, communication among HCWs and between HCWs and patients, coordination in the different TB care facilities, training for HCWs, and management and leadership in the TB programme. Reported patient-related factors comprise knowledge, attitudes, and behaviours about TB and its management and motivation towards keeping TB appointments or going to the clinic. Reported HCW-related factors include knowledge, attitudes, and behaviours about TB and its management. Using GRADE-CERQual, we had moderate confidence in most of our findings.

To our knowledge, this is the first QES on patients’ and HCWs’ perspectives on factors contributing to PTLFU. Strengths of the QES include searching multiple databases and having more than one reviewer involved at every stage of the synthesis including study selection, quality assessment, and data extraction. However, we did not search the grey literature and limited the studies to those published in English; thus, there is a possibility that we might have missed some relevant studies.

Our findings highlight some of the challenges that patients face when navigating the TB care cascade. Breakdown in communication between HCWs and patients concerning the next steps after diagnostic testing was cited as one of the obstacles to starting treatment [26,27]. This challenge needs to be addressed early in the TB care cascade otherwise it will also contribute to patients dropping out after they start treatment [28]. Other reported factors contributing to PTLFU are related directly to patients such as using alcohol and being an immigrant [27,29,30]. Stigma is still an impediment when it comes to patients starting treatment. This is similar to the findings from a QES on adherence to TB treatment [31]. Patients shy away from starting the medications since their relatives and people close to them will get to know their situation and view them as having human immunodeficiency virus/acquired immunodeficiency syndrome (HIV/AIDS). Long waiting times and frequent visits to the TB clinic also discouraged patients. Patients who work had to choose between going to the clinic and losing a day’s wages, and most opted to work in lieu of attending the clinic.

The lack of counselling about TB was reported as a factor affecting PTLFU. Counselling is important for TB care in that it enhances better patient outcomes by improving patients’ knowledge about TB and dispelling misconceptions about the disease [32,33]. HCWs can then clearly explain the meaning of test results and provide support as patients try to cope with the stress of the diagnosis. This is an opportunity to establish trust, correct misperceptions about TB, and encourage patients to start treatment.

Regarding the health system, we noted several concerns that may have affected HCW attitudes and interactions with patients leading to PTLFU [24,25]. For instance, staff shortages in the TB programme led to more work for those who were available. These shortages contributed to delays in patients receiving their test results and some did not return to the facility. Moreover, when the workload was high, HCWs could not spend adequate time with patients. Thus, patients did not receive information about what to do after collecting their test results and counselling on the importance of starting treatment. In addition, HCWs reported that contact details were frequently not verified during registration.

Limited financial support was also a barrier to the provision of TB services. HCWs reported that they did not receive enough financial support when it came to patient follow-up. Money for transportation and phone calls was not forthcoming. This resulted in HCWs becoming discouraged and in turn, they did not track patients who missed their clinic visits. Some HCWs went the extra mile in using their own money and resources for patient follow-up, but they were not reimbursed. This led HCWs to discontinue following up with patients leading to PLTFU.

National TB programmes should use routinely available data to address PTLFU. For example, TB programmes can monitor the proportion of notified TB cases that have bacteriologically confirmed disease and ensure that these patients are followed. Ultimately, placing people at the centre of care will enable HCWs to provide care holistically, knowing that apart from having TB, patients may face other challenges, such as food insecurity and lack of housing. Food insecurity and lack of housing are among the factors which may increase the risk of TB infection, disease, and poor clinical outcomes [34,35]. In addition, the recently published WHO ‘Guidance for national strategic planning for tuberculosis’ will facilitate the creation of comprehensive plans for TB at national and subnational levels [36]. The WHO guidance emphasizes the importance of high-level political commitment to ensuring adequate resources for TB care and prevention [2,36].

Of the five included studies in this QES, three were from one country [22,23,25]. We note this as a limitation because the factors identified in these studies may not apply to all settings. Four studies used an exploratory design. More qualitative studies should be done in different settings and using other designs such as ethnography to find out if the reasons across different settings and countries are similar. This information will be useful in informing policy regarding addressing PTLFU globally and in different settings.

### Review author reflexivity

The review author team has a range of research experience and expertise in clinical research and evidence synthesis in TB. This could have influenced their input in conducting the QES, therefore the following measures were factored in to moderate their influence. During the study selection process, the review authors resolved conflicts through discussion and aimed to achieve consensus as a team. Two review authors who were involved in data extraction and writing up the findings repeatedly discussed how their backgrounds may influence their data analysis and writing of the findings. They also questioned each other’s interpretations of the findings to assess their fit with the existing findings. The other review authors were also consulted to verify that the findings were reflections of the supporting data.

## Conclusion

In this QES, we found that multiple factors contributed to PTLFU, the main ones being the need to offer people-centred care and strengthen health systems. To be effective, interventions to address PTLFU should consider the concerns of patients and providers and place patients at the heart of care in the health system. Political commitment at the national level is needed to ensure adequate resources for addressing PTLFU and moving closer to the goal of ending TB.

## Supplementary Material

Supplemental Material

## Paper context

In the tuberculosis care cascade (model used to describe healthcare delivery), pre-treatment loss to follow-up has been shown to contribute substantially to patient losses, with subsequent high community transmission and mortality. Our qualitative evidence synthesis provides comprehensive evidence on contributors to pre-treatment loss to follow-up in adults with pulmonary TB and highlights areas to intervene. To achieve optimal care, it is vital to strengthen the healthcare system and offer people-centred care.
