# Supplementary material for: Factors contributing to pre-treatment loss to follow-up in adults with pulmonary tuberculosis: a qualitative evidence synthesis of patient and healthcare worker perspectives
Source: Glob Health Action. 2022 Dec 22;16(1):2148355. doi: 10.1080/16549716.2022.2148355 (PMC9788701; doi:10.1080/16549716.2022.2148355)
Supplement: Supplemental Material [file ZGHA_A_2148355_SM6300.docx]

**Supplemental material 3: Assessment of methodological limitations of included studies in the qualitative evidence synthesis of pre-treatment loss to follow-up**

| **Study ID** | **Domain** | | | | |
| --- | --- | --- | --- | --- | --- |
|  | **Were steps taken to increase rigour in the sampling?** | **Were steps taken to increase rigour in the data collected?** | **Were steps taken to increase rigour in the analysis of the data?** | **Were the findings of the study grounded in/ supported by the data?** | **Please rate the findings of the study in terms of their breadth and depth.** |
| **Squire 2005** | Yes, several steps were taken | Yes, several steps were taken | Yes, several steps were taken | Yes, a fairly thorough attempt was made | Yes, several steps were taken |
| **Sharma 2017** | Yes, several steps were taken | Yes, several steps were taken | Yes, a few steps were taken | Yes, a few steps were taken | Yes, several steps were taken |
| **Mwansa-Kambafwile 2020** | Yes, several steps were taken | Yes, several steps were taken | Yes, a fairly thorough attempt was made | Yes, a thorough attempt was made | Yes, several steps were taken |
| **Stalin 2020** | Yes, several steps were taken | Yes, a few steps were taken | Yes, several steps were taken | Yes, a few steps were taken | Yes, a few steps were taken |
| **Thomas 2020** | Yes, several steps were taken | Yes, several steps were taken | Yes, a fairly thorough attempt was made | Yes, a fairly thorough attempt was made | Yes, a fairly thorough attempt was made |
